# Sex Differences in Cerebral Blood Flow and Serum Inflammatory Cytokines and Their Relationships in Mild Traumatic Brain Injury

**DOI:** 10.3389/fneur.2021.755152

**Published:** 2022-01-26

**Authors:** Pinghui Zhao, Pingyi Zhu, Danbin Zhang, Bo Yin, Yu Wang, Nimo Mohamed Hussein, Zhihan Yan, Xiaozheng Liu, Guanghui Bai

**Affiliations:** ^1^Department of Radiology, The Second Affiliated Hospital and Yuying Children's Hospital of Wenzhou Medical University, Wenzhou, China; ^2^Department of Radiology, The First Affiliated Hospital, Zhejiang University School of Medicine, Hangzhou, China; ^3^Department of Neurosurgery, The Second Affiliated Hospital and Yuying Children's Hospital of Wenzhou Medical University, Wenzhou, China; ^4^China-USA Neuroimaging Research Institute, The Second Affiliated Hospital and Yuying Children's Hospital of Wenzhou Medical University, Wenzhou, China; ^5^Wenzhou Key Laboratory of Basic Science and Translational Research of Radiation Oncology, Wenzhou, China

**Keywords:** mild traumatic brain injury, serum inflammatory cytokines, cerebral blood flow, cognitive impairment, correlation analysis

## Abstract

This study aimed to investigate sex differences in cerebral blood flow (CBF) and serum inflammatory cytokines, as well as their correlations in patients with acute-stage mild traumatic brain injury (mTBI). Forty-one patients with mTBI and 23 matched healthy controls underwent 3D-pseudo-continuous arterial spin labeling imaging on 3T magnetic resonance imaging. The patients underwent cognitive evaluations and measurement of a panel of ten serum cytokines: interleukin (IL)-1I, IL-4, IL-6, IL-8, IL-10, IL-12, C–C motif chemokine ligand 2, interferon-gamma, nerve growth factor-beta (β-NGF), and tumor necrosis factor-alpha (TNF-α). Spearman rank correlation analysis was performed to evaluate the relationship between inflammation levels and CBF. We found that both male and female patients showed increased IL-1L and IL-6 levels. Female patients also demonstrated overexpression of IL-8 and low expression of IL-4. As for CBF levels, three brain regions [the right superior frontal gyrus (SFG_R), left putamen, and right precuneus] increased in male patients while three brain regions [the right superior temporal gyrus (STG_R), left middle occipital gyrus, and right postcentral (PoCG_R)] decreased in female patients. Furthermore, the STG_R in female controls was positively correlated with β-NGF while the right PoCG_R in female patients was negatively correlated with IL-8. In addition, compared with male patients, female patients showed decreased CBF in the right pallidum, which was negatively correlated with IL-8. These findings revealed abnormal expression of serum inflammatory cytokines and CBF levels post-mTBI. Females may be more sensitive to inflammatory and CBF changes and thus more likely to get cognitive impairment. This may suggest the need to pay closer attention to the female mTBI group.

## Introduction

Traumatic brain injury (TBI), which is often referred to as a silent epidemic, is defined as a neurotrauma caused by a mechanical force applied to the head (1). TBI is a significant public health issue with an increasing worldwide incidence (2). Mild TBI (mTBI) comprises more than 80% of all TBI cases (3) and often presents diagnostic and therapeutic challenges (4), especially in asymptomatic patients with mTBI-indicative imaging characteristics. Sex differences after mTBI has become a popular research topic recently. Animal experiments have claimed that compared with males, females have a higher survival rate and cognitive function post-TBI (5, 6), suggesting that female sexuality plays a neuroprotective role to some extent. In contrast, multiple human observational studies reported poorer outcomes in females after TBI (7). The role of sex differences in mTBI in humans is indeed an interesting topic to explore.

Secondary brain injury such as hypotension, hypoxia, fever, and intracranial hypertension, can impair brain tissue oxygenation and cerebral blood flow (CBF) (8). However, owing to the heterogeneity and complexity of the mechanisms underlying traumatic brain injury (9), CBF levels differ across patients with mTBI. 3D-pseudo-continuous arterial spin labeling technology allows for non-invasive and quantitative measurement (10, 11) and can directly reflect CBF changes, which yields a wide range of potential clinical application. Cunningham et al. (12), Kawai et al. (13), and Rostami et al. (14) have assessed CBF changes on moderate and severe TBI. Studies with respect to cognitive function, functional connections, and cortical thickness (15–18) in response to mTBI have also been focused on, some of which paid special attention to sex differences. For example, Bai et al. (16) suggested that increased CBF in the posterior parietal cortex could predict worse cognitive performance in male patients, which indicated a protective effect against neuropsychological impairments among females. However, the CBF changes in these brain regions in response to sex differences remain unclear, and there have been inconsistent results on mTBI. Therefore, further analysis is necessary.

Extensive evidence suggests that immune system activation and inflammation are central mediators of secondary injury after TBI (19), and emerging data showing that blood-based TBI biomarkers have the potential to predict the TBI outcome (20). Previous studies on biomarkers usually focused on neuronal cell body injury (UHL-L1, NSE) (21, 22), astroglial injury (GFAP, S100B) (23, 24), neuronal cell death (alpha II-spectrin breakdown products) (25, 26), and post-injury neurodegeneration (Tau) (27). However, there has been relatively little discussion on inflammatory cytokines in patients with mTBI. Within a few minutes of TBI trauma, various inflammatory cytokines are released into the central nervous system and peripheral blood (20, 28). A growing body of evidence supports the idea that limiting inflammatory response can improve the outcome of many diseases (29), including autoimmune diseases and multi-system trauma.

Serum biomarkers are currently being used clinically to diagnose some diseases, for instance, such as troponin in myocardial infarction, brain naturetic peptide in congestive heart failure, and amylase/lipase in pancreatitis (30). In addition, reverse phase protein microarray has identified modulation of inflammatory markers, especially interleukins in TBI studies (30). Although no single or panel of biomarkers of brain injury has unequivocally passed the specificity test across insults, these previously identified markers may help guide therapy in a variety of clinical settings (31), which leads us to the idea that there are some important members of the inflammatory cytokines possibly associated with mTBI. Trauma-related serum inflammatory cytokines, which include tumor necrosis factor-alpha (TNF-α), interleukin (IL)−1β, IL-2, IL-4, IL-6, IL-8, IL-18, IL-7, and IL-10, which may exert anti-inflammatory effects (32–34). However, the pro-inflammatory and anti-inflammatory effects in cellular damage and protection remain unclear, and most of them are focused on moderate-to-severe TBI (30). Our team previously studied 52 patients with mTBI and observed an increase in IL-1β, IL-6, and C–C motif chemokine ligand 2 (CCL2) levels over a period of 3 months (35). Based on these findings, we want to further explore the sex differences of inflammatory cytokines in the acute phase and evaluate their effects on cognitive impairments in combination with changes of CBF levels.

This study aimed to determine how the serum inflammatory cytokines and cerebral blood flow changed with sex differences post-mTBI. Additionally, we aimed to further evaluate their clinical utility by analyzing the correlation between these two factors. This may provide some valuable information for facilitating early clinical prediction and targeted treatment of mTBI.

## Materials and Methods

### Participants

We enrolled patients with mTBI who visited the Second Affiliated Hospital of Wenzhou Medical University Emergency Department between August 2016 and June 2017. The inclusion criteria were based on the principles of the World Health Organization's Collaborating Centre for Neurotrauma Task Force as follows (36): (i) an initial Glasgow Coma Scale (GCS) score of 13–15; (ii) loss of consciousness (LOC) episode for < 30 min, post-traumatic amnesia (PTA) < 24 h, and other transient neurological abnormalities, including focal signs, seizures, and intracranial lesions not requiring surgery; (iii) mTBI onset within the previous 1 week; (iv) age >18 years; and (v) agreement to communicate by telephone or email and to return to the hospital for follow-up assessment. During initial screening, priority was given to patients with mTBI who were already examined and were negative for chest and abdominal abnormalities. At the same time, there were no abnormalities on CT scans of all the patients. The exclusion criteria were as follows: (i) LOC > 30 min, PTA > 24 h, and a GCS score < 13 after 30 min; (ii) history of TBI, neurological disorders, chronic psychiatric conditions, and substance abuse; (iii) intracranial hemorrhage, hematoma formation, cerebral hernia, etc. (abnormal CT and MRI findings); (iv) intubation and/or presence of skull fracture and sedation use; (v) mTBI manifestation as a complication of other injuries (e.g., systemic, facial, or spinal cord injuries); (vi) other problems (e.g., psychological trauma, language barrier, or coexisting medical conditions); and (vii) mTBI caused by penetrating craniocerebral injury.

We enrolled a total of 41 patients (17 females and 24 males) with mTBI who completed the initial assessments. We recruited 23 sex-, age-, and education-matched healthy participants (13 females and 10 males) without psychiatric disorders from local imaging research facilities. All the participants were right-handed based on the Edinburgh Handedness Inventory (37). All participants provided in-person written informed consent. This study was approved by the Research Ethics Committee of the Second Affiliated Hospital of Wenzhou Medical University and conducted in accordance with the Declaration of Helsinki.

### Neuropsychological Tests

All the participants underwent comprehensive cognitive assessments within 48 h of blood sample collection and MRI acquisition. This regimen was based on a previous study on TBI-related brain structural alterations (38). The cognitive assessments performed were as follows: (i) Trail Making Test Part A and Digit Symbol Coding score from the Wechsler Adult Intelligence Scale-III (WAIS-III) for measuring cognitive information processing speed; (ii) Forward Digit Span and Backward Digit Span from the WAIS-III to assess working memory and executive function (39); (iii) Language Fluency Test to assess verbal fluency, including language ability and executive function (40); (iv) Beck Depression Inventory-II to assess depression severity (41); (v) PTSD Checklist-Civilian Version (PCL-C) (42); and (vi) Fatigue Severity Scale (FSS) (43) (FSS) and Insomnia Severity Index (ISI) (44). Additionally, the Rivermead Post-Concussion Symptom Questionnaire (RPCS) was used to measured the post-concussive symptoms (45).

### Serum Collection and Separation

Serum samples were collected within 48 h after injury in the patients' group and depending on the controls' will within the same time period, usually in the early morning at 07:00–08:00 A.M. Before sample collection, participants were not allowed to take medicine or eat food for almost about 8 h. Take 5 mL of subjects' fasting peripheral blood in BD vacuum flask (red top) (Cat#367812) (preservative-free, 8 mL). The blood was first left standing at room temperature for 30–60 min. After blood coagulation, the serum was then separated at room temperature (4,000 rpm, 5 min). The serum was injected into 2 mL cryotube at the rate of 500 μL per sample, and labeled. All the sample tubes were transferred to −80°C for subsequent detection of inflammatory factors. Blood samples containing inflammatory cytokines were processed using the Luminex kit (Luminex, Austin, TX, USA) following the manufacturer's instructions (46). A fluorescence detection system was used to simultaneously detect the binding of each protein onto microspheres, which allowed determination of several analytes within a single sample. The intra- and inter-assay coefficients of variation for Luminex quantification were < 20 and 25%, respectively, and the detection limit was < 0.01 pg/mL. Further, the detection limit was < 0.01 pg/mL. We analyzed inflammatory cytokines that reflect different aspects of TBI injury, including pro-inflammatory cytokines (IL-1β, IL-6, and IL-12), anti-inflammatory cytokines (IL-4 and IL-10), CCL2, IL-8, TNF-α, and interferon-gamma.

### Image Acquisition

After acute head injury, all the patients underwent non-contrast CT scans (LightSpeed VCT, GE Healthcare, Milwaukee, WI, USA) scans to exclude other intracranial lesions. All MRI scans were obtained on a 3.0T MR system (Discovery MR750 3.0T; GE Healthcare) equipped with a custom-built head holder for the prevention of head movement. The following scan parameters were chosen based on previous studies and the Equipment Parameter Recommendation Guide (47): time of repetition (TR) = 5,046 ms, time of echo (TE) = 11 ms, slice thickness = 3 mm, field-of-view (FOV) = 24 × 24 mm^2^, labeling time =1.5 s, and post-labeling delay = 2,000 ms. Moreover, a separate M0 image used for CBF quantification was obtained without magnetization preparation. Subsequently, we obtained high-resolution, sagittal, three-dimensional T1 images using the following parameters: TR = 2,100 ms, TE = 3 ms, slice thickness = 1 mm, FOV = 25.6 × 25.6 mm^2^, and flip angle = 9°. Additionally, axial susceptibility-weighted imaging (SWI) was performed to exclude micro-bleeding using the following parameters: TR = 37.8 ms, TE = 25 ms, flip angle = 15°, slice thickness = 2 mm, slices = 70, FOV = 230 × 230 mm^2^, and matrix size = 512 × 512 mm^2^.

### 3D-Arterial Spin Cerebral Blood Flow Analysis

The images were processed at the Wellcome Centre for Human Neuroimaging, UCL Queen Square Institute of Neurology, London, UK) on the MATLAB platform (R2013a; MathWorks, Natick, MA, USA). First, the images were registered to the standard template (NIST, MNI, McGill University, Montreal, QC, Canada). This was followed by resetting of arterial spin-labeling (ASL) images and centering of the structural images on the image matrix. Cerebral blood flow maps (units: mL/100 g tissue/min) were generated using batch scripts available on ASLtbx (48). T1 images were segmented into gray matter, white matter, and CSF tissue probability maps (TPMs); furthermore, we generated a binary mask to extract the mean CBF. Next, the ASL images were remapped onto individual T1 spaces. Finally, the mean CBF maps were smoothened using a 6-mm full-width-at-half-maximum isotropic Gaussian filter.

### Statistical Analyses

Statistical analyses were performed using SPSS (version 23, IBM, Armonk, NY, USA) and Prism 7 (GraphPad Software, San Diego, CA, USA). For all continuous variables, the normality of data distribution was examined using the Shapiro-Wilk test. Between-group comparisons of normally and non-normally distributed data were performed using the independent two-sample *t*-test and the Mann-Whitney *U*-test, respectively. Categorical variables were assessed using the chi-square test. Statistical significance was set at *p* < 0.05. For brain regions with varying mean CBF measurements, we extracted values using REST (http://restfmri.net/forum/RESTplusV1.2). Spearman rank correlation analysis was performed to determine correlations between serum cytokine levels and CBF levels in patients with mTBI. Here, serum levels of inflammatory factors, age, sex, and educational level were considered as independent variables while CBF was the dependent variable.

## Results

### Demographics and Neuropsychological Data

We enrolled 41 patients (17 females and 24 males) with mTBI, with a mean age of 37.4 ± 2.5 years and 23 controls (13 females and 10 males) with a mean age of 36.2 ± 2.7 years. There were no between-group differences in age, education level, and sex (*p* > 0.05). Table 1 presents the demographic and clinical characteristics of both groups. All the patients with mTBI had an initial GCS score of 15. The causes of injury included acceleration/deceleration (58.5%), assault (19.5%), falls (19.2%, ground-level falls and falls from height), and direct-impact blows to the head (2.0%).

**Table 1:** Summary of demographics and neuropsychological information for patients with mTBI and control subjects.

	**mTBI** **(***n*** = 41)**	**Controls** **(***n*** = 23)**	***P-*** **value**
**Demographic**			
Age (y)	37.4 ± 2.5	36.2 ± 2.7	0.120^a^
Gender (M/F)	24/17	10/13	0.226^a^
Education (Y)	8.4 ± 4.0	9.1 ± 6.3	0.725^a^
**Neuropsychological tests**			
TMT-A (s)	64.1 ± 44.3	55.8 ± 32.9	0.409^b^
RPCS	10.1 ± 7.5	2.4 ± 2.9	**<0.001** ^*^ ^b^
PCL-C	24.6 ± 6.1	17.0 ± 0.0	**<0.001** ^*^ ^b^
DCS	33.5 ± 16.2	38.8 ± 17.9	0.142^b^
FDS	7.8 ± 1.5	7.8 ± 1.7	0.657^b^
BDS	4.0 ± 1.5	4.3 ± 1.9	0.359^b^
LF	10.3 ± 2.5	17.4 ± 5.7	**<** 0.001^*^^b^
Beck	4.3 ± 4.0	0.03 ± 0.2	**<** 0.001^*^^b^
FSS	9.8 ± 4.1	9.0 ± 0.0	0.127^b^
ISI	6.8 ± 5.9	1.5 ± 2.2	**<0.001** ^*^ ^b^
**MTBI severity n (%)**			
Loss of conscious	36 (87.8%)	NA	
Post-traumatic amnesia	5 (12.2%)	NA	
GCS = 15	41 (100%)	NA	
GCS = 13, 14	0 (0%)	NA	
**Causes for mTBI n (%)**			
Acceleration/deceleration	24 (58.5%)	NA	
Ground level fall	3 (7.0%)	NA	
Fall from height	5 (12.2%)	NA	
Assaults	8 (19.5%)	NA	
Direct impact blow to head	1 (2.0%)	NA	

a *Chi-square test*;

b *two-independent-sample t-test; TMT-A, Trail-Making Test Part A; RPCS, Rivermead Post-Concussion Symptom Questionnaire; PCL-C, Post-traumatic stress disorder Checklist civilian; DSC, Digit Symbol Coding; FDS, Forward Digit Span Task; BDS, Backward Digit Span Task; LF, Language Fluency Test; Beck, Beck Depression Inventory; FSS, Fatigue Severity Scale; ISI, Insomnia Severity Index; GCS, Glasgow Coma Scale; mTBI, mild traumatic brain injury; NA, non-available*.

* *Significant at p < 0.05*.

Compared with all the controls, the patients with mTBI performed worse in the RPCS, PCL–C, LF, Beck, and ISI (*p* < 0.001, Table 1). Compared with female controls, female patients performed worse on almost all neuropsychological tests except for the FSS (*p* < 0.01 for all tests, Table 2). Compared with male controls, male patients performed worse on the RPCS, PCL–C, LF, Beck, FSS, and ISI (*p* < 0.05, Table 2).

**Table 2:** Neuropsychological tests outcomes in patients with mTBI and healthy controls.

**Neuropsychological tests**	**Males**		***P*** **-value**	**Females**		***P*** **-value**
	**Patient** **(***n*** = 24)**	**Control** **(***n*** = 17)**		**Patient** **(***n*** = 10)**	**Control** **(***n*** = 13)**	
TMT-A	74.2 ± 12	48.8 ± 4.5	0.250	51.6 ± 4.5	33.2 ± 4.4	0.002^*^
RPCS	9.2 ± 1.1	1.1 ± 0.3	**<0.001** ^*^	11.3 ± 1.6	3.5 ± 0.7	**<0.001** ^*^
PCL-C	23.2 ± 0.7	17.0 ± 0.0	**<0.001** ^*^	25.7 ± 1.4	17.0 ± 0.0	**<0.001** ^*^
PCS	36.9 ± 3.7	42.9 ± 3.6	0.242	34.5 ± 2.9	52.3 ± 2.8	**<0.001** ^*^
FDS	7.9 ± 0.4	7.9 ± 0.3	0.982	7.9 ± 0.3	9.3 ± 0.3	**0.006** ^*^
BDS	3.8 ± 0.3	3.9 ± 0.2	0.656	3.8 ± 0.3	5.5 ± 0.5	**0.004** ^*^
LF	10.5 ± 1.0	20.8 ± 1.4	**<0.001** ^*^	10.4 ± 1.2	18.6 ± 1.4	**<0.001** ^*^
Beck	3.9 ± 0.5	0.0 ± 0.0	**<0.001** ^*^	5 ± 1.2	0.0 ± 0.0	**<0.001** ^*^
FSS	11.9 ± 1.6	9.0 ± 0.0	**0.043** ^*^	9.5 ± 0.4	9.0 ± 0.0	0.186
ISI	7.1 ± 0.9	1.0 ± 0.5	**<0.001** ^*^	7.1 ± 1.6	1.9 ± 0.4	**0.029** ^*^

*TMT-A, Trail-Making Test Part A; RPCS, Rivermead Post-Concussion Symptom Questionnaire; PCL-C, Post-traumatic stress disorder Checklist civilian; DSC, Digit Symbol Coding; FDS, Forward Digit Span Task; BDS, Backward Digit Span Task; LF, Language Fluency Test; Beck, Beck Depression Inventory; FSS, Fatigue Severity Scale; ISI, Insomnia Severity Index; mTBI, mild traumatic brain injury*.

* *Significant at p < 0.05*.

### Serum Cytokine Levels

Compared with male controls, male patients showed significantly increased levels of IL-1β (*p* = 0.038) and IL-6 (*p* = 0.004) in the acute phase. On the contrary, compared with female controls, female patients showed significantly decreased levels of IL-4 (*p*=0.007), as well as increased levels of IL-1β (*p* = 0.026), IL-6 (*p* = 0.009), and IL-8 (*p* = 0.025). Table 3 presents the descriptive statistics for serum cytokine levels.

**Table 3:** MTBI inflammatory cytokines detection outcomes in patients with mTBI and healthy controls.

**Serum cytokine**	**Males**		***P*** **-value**	**Females**		***P*** **-value**
	**Patient** **(***n*** = 24)**	**Control** **(***n*** = 17)**		**Patient** **(***n*** = 10)**	**Control** **(***n*** = 13)**	
β-NGF	6.4 ± 11.3	3.9 ± 4.0	0.373	10.1 ± 22.7	2.8 ± 1.1	0.859
CCL2	225.7 ± 85.1	247.2 ± 48.3	0.163	228.7 ± 70.6	236.4 ± 85.4	0.965
IL-1β	3.2 ± 1.3	1.5 ± 0.6	**0.038** ^*^	7.5 ± 2.6	2.7 ± 0.5	**0.026** ^*^
IL-4	13.6 ± 19.2	10.0 ± 7.8	0.983	7.2 ± 13.6	16.0 ± 8.1	**0.007** ^*^
IL-6	2.6 ± 3.5	0.8 ± 0.3	**0.004** ^*^	4.2 ± 2.6	1.2 ± 0.3	**0.009** ^*^
IL-8	9.7 ± 5.0	7.5 ± 1.8	0.197	9.8 ± 4.2	6.4 ± 2.4	**0.025** ^*^
IL-10	0.8 ± 0.7	1.1 ± 0.9	0.512	0.5 ± 0.5	0.7 ± 0.4	0.088
IL-12	37.0 ± 67.4	26.3 ± 22.4	0.330	31.5 ± 46.1	43.1 ± 73.2	0.410
INF-γ	24.1 ± 67.8	15.4 ± 13.7	0.226	23.1 ± 28.8	14.7 ± 8.2	0.893
TNF-α	3.4 ± 2.9	2.5 ± 1.5	0.626	3.2 ± 3.7	2.7 ± 0.9	0.315

*Bold indicates a statistically significant difference; mTBI, mild traumatic brain injury; β-NGF, nerve growth factor beta; CCL2, C–C motif chemokine ligand 2; IL-1β, interleukin-1 beta; IL-4, interleukin 4; IL-6, interleukin 6; IL-8, interleukin 8; IL-10, interleukin 10; IL-12, interleukin 12; INF-γ, interferon gamma; TNF-α, tumor necrosis factor alpha*.

* *Significant at p < 0.05*.

### Differences in Changes in Cerebral Blood Flow Levels

**Figure 1:**
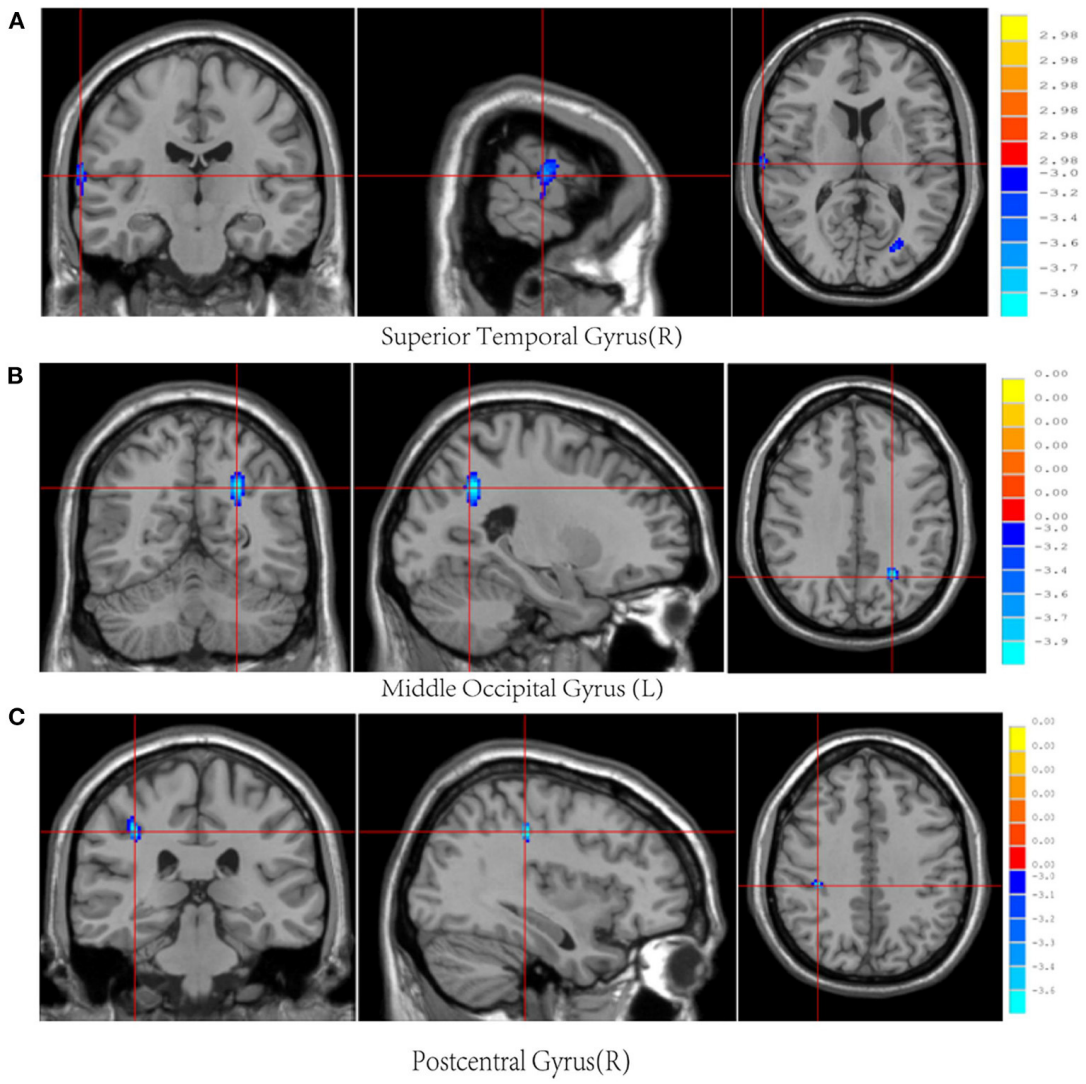
Results of brain region changes in mTBI female patients compared with female controls. The CBF of female patients in right superior temporal gyrus **(A)**, left middle occipital gyrus **(B)**, and right postcentral gyrus **(C)** showed decreased perfusion levels compared with female controls. mTBI, mild traumatic brain injury; CBF, cerebral blood flow.

**Figure 2:**
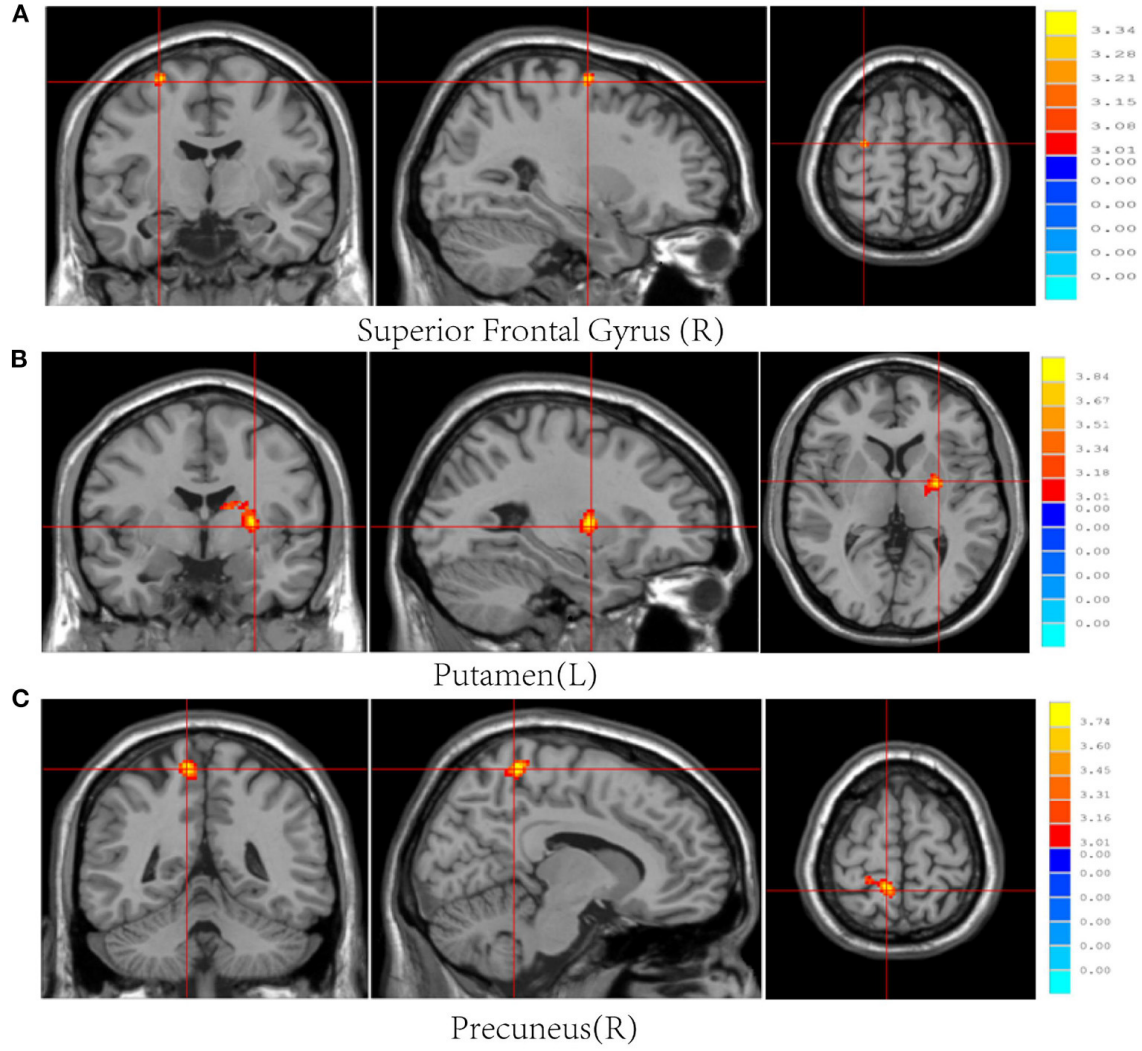
Results of brain region changes in mTBI male patients compared with male controls. The CBF of male patients in right superior frontal gyrus **(A)**, left putamen **(B)**, and right precuneus **(C)** showed increased perfusion levels compared with male controls. mTBI, mild traumatic brain injury; CBF, cerebral blood flow.

**Figure 3:**
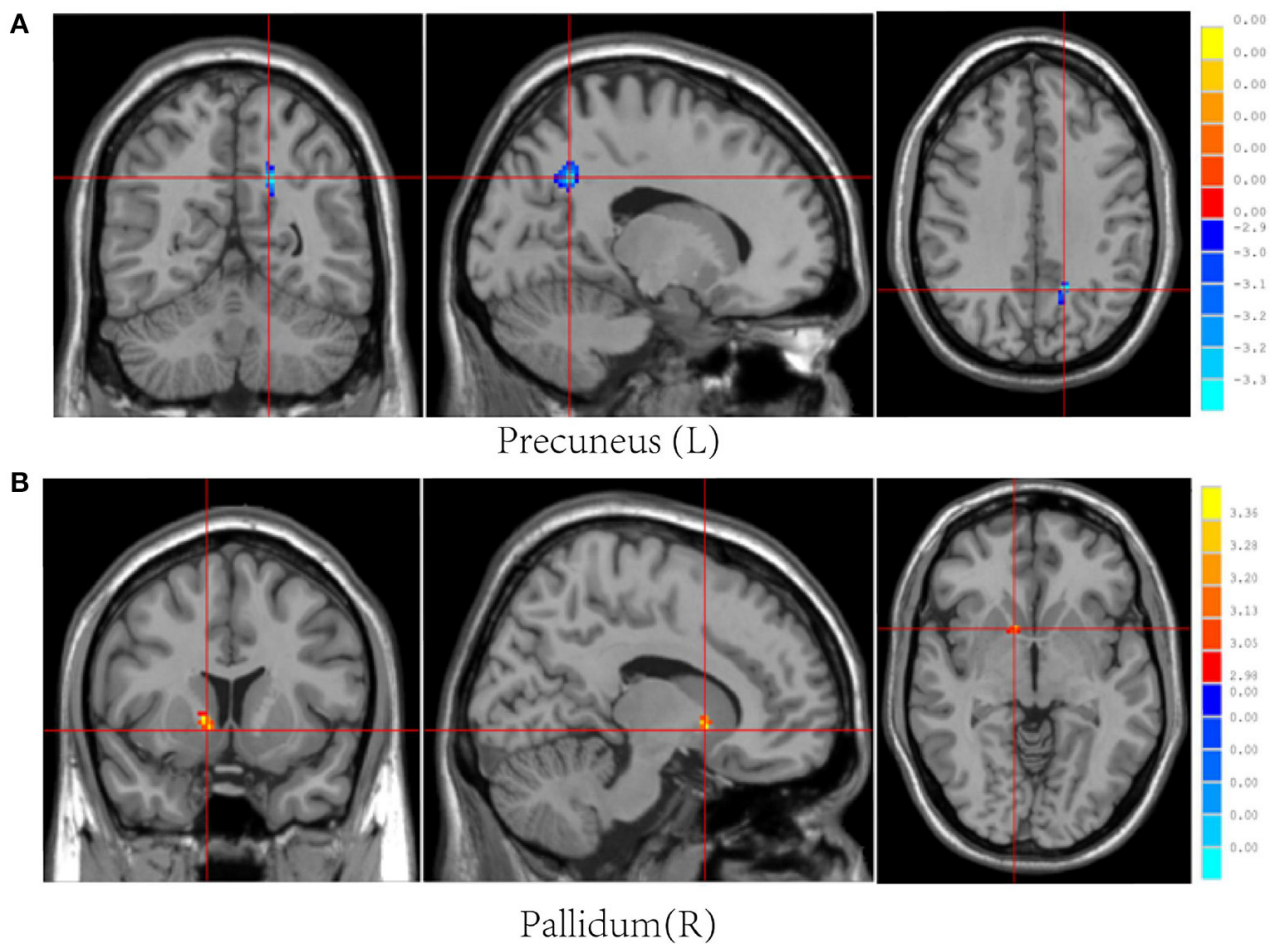
Results of brain region changes in mTBI male patients compared with female patients. The CBF of male patients in left precuneus **(A)** is lower than female patients, while the right pallidum **(B)** showed increased perfusion levels. mTBI, mild traumatic brain injury; CBF, cerebral blood flow.

Post-mTBI CBF changes occurred in different brain regions in male and female patients. Compared with female controls, female patients showed three decreased CBF regions in the right superior temporal gyrus (STG_R), left middle occipital gyrus (MOG_L), and right postcentral gyrus (PoCG_R) (Table 4 and Figure 1). In contrast, compared with male controls, male patients showed three increased CBF regions in the right superior frontal gyrus (SFG_R), left putamen, and right precuneus (Figure 2). Additionally, compared with male patients, female patients showed lower and higher CBF levels in the pallidus and left precuneus, respectively (Figure 3).

**Table 4:** The significantly altered CBF brain regions between the patients with mTBI and healthy controls.

**Brain regions**	**CBF units: mL/100 g tissue/min**	**Peak MNI coordinate**			***t*** **-value**	**number of voxels**
		**x**	**y**	**z**		
**Decreased CBF in female patients vs. female controls**						
Superior Temporal Gyrus(R)	42.676	68	−18	12	−3.662	71
Middle Occipital Gyrus (L)	57.053	−24	−58	36	−4.158	103
Postcentral Gyrus(R)	52.662	36	−30	40	−3.710	43
**Increased CBF in male patients vs. male controls**						
Superior Frontal Gyrus (R)	64.030	−14	26	58	3.343	28
Putamen(L)	48.713	−28	−2	4	4.003	375
Precuneus(R)	58.787	10	−46	66	3.889	202
**Decreased CBF in male patients vs. female patients**						
Precuneus (L)	49.127	−16	−56	36	−3.421	77
**Increased CBF in male patients vs. female patients**						
Pallidum(R)	55.917	12	10	−4	3.433	34

*CBF, cerebral blood flow; mTBI, mild traumatic brain injury; MNI, Montreal Neurological Institute; L, left; R, right; x, y, z, coordinates of peak voxel*.

### Correlation Between Cerebral Blood Flow and Serum Inflammatory Factors

**Figure 4:**
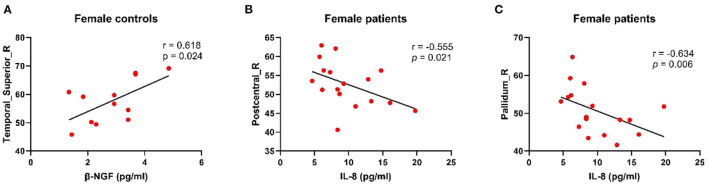
Results of correction between CBF and serum inflammatory factors. **(A)** Decreased CBF perfusion in the right superior temporal gyrus in female controls was associated with decreased β-NGF, which was positively correlated. **(B)** In female patients, CBF level in the right posterior central gyrus was negatively correlated with inflammatory factor IL-8. **(C)** In the right pallidum, the higher the IL-8 concentration is, the lower the blood volume level in that area. CBF, cerebral blood flow; β-NGF, nerve growth factor beta; IL-8, interleukin 8.

Figure 4 presents the relationships between CBF levels and serum inflammatory cytokines. There was a positive correlation between decreased CBF levels in the STG_R with β-NGF downregulation in female controls (*r* = 0.618, *p* = 0.024). Moreover, CBF levels in the PoCG_R were negatively correlated with IL-8 levels in female patients (*r* = −0.555, *p* = 0.021). Specifically, compared with the male patients, female patients showed a negative correlation of CBF levels in the pallidus with IL-8 levels (*r* = −0.634, *p* = 0.006).

## Discussion

This study investigated post-mTBI changes in CBF levels and serum inflammatory cytokines, as well as their correlations. We observed some differences in serum inflammatory cytokines and CBF levels post-mTBI between the two sexes. To the best of our knowledge, this is the first study to investigate sex differences in inflammatory cytokines expression and CBF levels in patients with mTBI. Using supplementary neurocognitive tests, we further explored relationships among these factors, which may provide some valuable information for facilitating early clinical prediction and targeted treatment of mTBI.

In the acute phase, we discovered some sex differences in inflammatory cytokines post-mTBI. We found that both male and female patients had increased IL-1β and IL-6 levels, which was consistent with our previous team works (35). It has been reported that pattern recognition receptors can induce the transcription of inflammatory cytokines, including TNF-α, and interleukins (such as IL-1β, IL-6, and IL-18), which orchestrate a systemic inflammatory response (49). IL-6 and IL-1β have been found to be significantly elevated in the acute phase after mTBI (50). Literature suggested that TNF-α, IL-1β, and IL-8 are released early (within hours to 2 days) after the traumatic injury, reflecting their putative roles as inflammatory response initiators (51). Increased expression of IL-1β and IL-6, which are classic pro-inflammatory cytokines, are suggestive of post-mTBI inflammatory responses and presents significant danger to patients. IL-1β has similar functions as TNF-α, which is associated with mTBI-related cognitive impairment in acute working memory (35). Another report found that neutralizing IL-6 can reduce the neuroinflammatory response, significantly reduce brain damage, and completely eliminate the loss of motor coordination ability after mTBI (52). Multiple neuropsychological scale abnormalities in the patient group suggested that elevated levels of IL-1β and IL-6 may indeed have an impact on cognition in patients with mTBI.

However, in our female patients, mTBI-mediated neuroinflammation was characterized by high and low IL-8 and low IL-4 expression, respectively. Previous studies have reported that IL-8, excreted by monocytes and endothelial cells, can induce both regenerative and degenerative processes, and numerous studies have shown that IL-8 can aggravate TBI-induced damage (53–55). Serum IL-8 levels can be used to assess TBI severity with IL-8 overexpression often predicting a poor prognosis for patients with TBI (54). IL-4, which is secreted by T helper 2 cells, is crucially involved in higher brain functions, including spatial memory, learning, and neurological disorders (56). Studies on stroke and multiple sclerosis have shown that IL-4 has a protective factor against spinal cord injury (57). A study on IL-4 knockout mice reported that IL-4 injection into the spinal cord induced microglial and macrophagic expression, which promotes anti-inflammatory effects (58). In our study, high and low IL-8 and low IL-4 expression, respectively, indicated greater mTBI severity in female patients, which suggests that females have a greater risk of post-trauma inflammatory cytokine imbalance and worse prognosis compared with males.

Although there is a lot of literature pointing to the role of individual inflammatory cytokiness in TBI, previous reviews also concluded that “no biomarker has consistently demonstrated the ability to predict post-concussive syndrome after mTBI” (59) and “the discriminative power of the biomarkers alone was limited” (60), which prompted us to do more research in depth.

There has been increasing research on brain microcirculation in patients with TBI (61), however, sex differences in the regulation of CBF and serum inflammatory cytokines remain unclear. Generally, in our present study, we found that there were significant differences in CBF levels between different sex groups. In female patients with mTBI, there was a decrease in CBF levels in the STG_R, MOG_L, and PoCG_R. In contrast, in male patients, there were increased CBF levels in the SFG_L, left putamen, and right precuneus. The precuneus and frontal cortex are important parts of the default mode network (DMN) (62). Previous studies have shown that after mTBI injury, the balance between DMN and other brain regions seems to be partially disrupted, which may lead to cognitive fatigue (61). Increased CBF in the precuneus and frontal cortex may be a potentially compensatory response, suggesting protection from the DMN against more severe cognitive impairment in males. The putamen, a component of the striatum, receives input information from the prefrontal cortex, and plays a larger role in working memory (63). The increased CBF levels in these three regions in the male patients may represent a neuroprotective mechanism where the brain attempts to maintain its energy balance during the acute mTBI phase to compensate for decreased ATP production in the damaged regions (16). STG is known as a region associated with many auditory language tasks, involving the integration of information from auditory and visual tasks (64). PoCG (Brodmann area 1,2,3) is the primary sensory receptive area of touch and kinesthesia, and a correlation has been found between PoCG volume and hallucination severity (65). The occipital cortex is the visual perception and processing center, which responsible for the multi-sensory integration of visual, auditory, and tactile information (66). These three decreased CBF levels in females patients may indicate inhibition or damage of neuroprotective mechanisms. One major biological difference between females and males may be the cyclical changes in the steroid estrogen and progestin levels in females during their menstrual periods (67). It is thus possible that normal physiological post-mTBI in these gonadal hormones may be the basis for the relatively poor outcomes observed.

Our team previously assessed the relationship between CBF levels and neuropsychological scales (16). In this present study, we found that both female and male patients with mTBI presented complaints on self-reported symptomatology; however, there were sex differences in neuropsychological performance and CBF changes with sex displaying a salient modulatory. Consequently, we conducted further research on post-mTBI CBF levels, inflammatory factor levels, and neuropsychological tests.

In the current study, we found that female patients exhibited a negative correlation between CBF levels in the PoCG_R with IL-8 levels. Furthermore, female controls showed a positive correlation between CBF levels in the STG_R with β-NGF expression. Although PoCG received little attention in the brain imaging literature, some studies also found PoCG abnormalities in patients with degenerative diseases (65), and some also showed that PoCG was the main sensory receptive area (68). In female patients, the negative correlation between CBF levels in the PoCG_R with IL-8 levels may affect their spatial-perception ability, which results in dizziness and fatigue. In this study, neurocognitive tests revealed that female patients were more prone to fatigue. The STG is associated with numerous auditory language tasks, including speech and hearing, as well as comprehension of metaphors (69). Studies have reported the neuroprotective and regenerative effects of β-NGF (70); moreover, NGF gene therapy has been shown to selectively improve post-mTBI cognitive function (71). Therefore, normal NGF expression for stimulating the central nervous system after TBI is crucial for cell survival (72). In female controls, there was a positive correlation of CBF levels in the STG_R CBF with β-NGF levels. This positive correlation may disappeared, and neuropsychological tests revealed that female patients had significantly impaired language fluency. This suggests that decreased β-NGF expression and CBF in the STG may have synergistic effects, which reflect metabolic abnormalities and language deficits, and therefore lead to more severe injury.

In this study, female patients also showed a negative correlation between CBF levels in the pallidus with IL-8 levels. Pallidus is part of the basal ganglia circuit connecting the striatum and the subthalamic nucleus, and is the most important deep central brain structure (73, 74). It has been reported that Parkinson's-like pathology observed in TBI may be related to basal ganglia dysfunction (75), and this area has been shown to be highly sensitive to injuries following focal brain trauma in humans. Deep brain stimulation of the pallidus has been shown to improve depressive symptoms in patients with Parkinson's disease (76). However, as IL-8 levels increased, CBF to the pallidus decreased, which reduced the function of this region and caused corresponding neurocognitive degeneration.

This study has several limitations. First, we only examined changes in serum inflammatory cytokines and CBF perfusion levels during the acute phase. Given that microglial cells may remain active in the central nervous system for long periods, even months or years, there is a need for a longitudinal study on sex differences in post-mTBI changes in inflammatory cytokines and CBF levels. Second, this study had a relatively small sample size, which could be attributed to missing data resulting from loss to follow-up. Future studies should enroll a higher number of participants with regular follow-ups. Finally, a particular cytokine may have a variety of different functional roles, depending on when it is released after the initial traumatic event, other co-releasing factors, and different injury mechanisms. Because mTBI has a complex pathophysiology, correlation analysis of inflammatory factors and CBF levels alone cannot accurately reflect the underlying mTBI mechanisms. Therefore, further research and clarification are warranted.

## Conclusions

This study found sex differences in post-mTBI changes in serum levels of inflammatory cytokines and CBF levels in specific brain regions. Furthermore, mTBI-mediated neuroinflammation was characterized by abnormal expression of serum inflammatory factors. In females, IL-4 underexpression and IL-8 overexpression might lead to poor prognosis. The increased CBF in male patients may be a compensatory effect, which plays a cognitive protective role in males. However, females may be more sensitive to inflammatory changes and more likely to have cognitive impairment. This suggests the need to pay closer attention to the female patients with mTBI.

## Data Availability Statement

The raw data supporting the conclusions of this article will be made available by the authors, without undue reservation.

## Ethics Statement

The studies involving human participants were reviewed and approved by Research Ethics Committee of the 2nd Affiliated Hospital of Wenzhou Medical University. The patients/participants provided their written informed consent to participate in this study.

## Author Contributions

PZhao and PZhu contributed equally to this paper. All authors listed have made a substantial, direct, and intellectual contribution to the work and approved it for publication.

## Funding

This research was supported by the National Natural Science Foundation of China (Grant No. 81771914), Natural Science Foundation of Zhejiang Province (nos. LY19H180003) and Wenzhou Science and Technology Bureau in China (No. Y20180184).

## Conflict of Interest

The authors declare that the research was conducted in the absence of any commercial or financial relationships that could be construed as a potential conflict of interest.

## Publisher's Note

All claims expressed in this article are solely those of the authors and do not necessarily represent those of their affiliated organizations, or those of the publisher, the editors and the reviewers. Any product that may be evaluated in this article, or claim that may be made by its manufacturer, is not guaranteed or endorsed by the publisher.
